# Supplementary material for: COZOID: contact zone identifier for visual analysis of protein-protein interactions
Source: BMC Bioinformatics. 2018 Apr 6;19:125. doi: 10.1186/s12859-018-2113-6 (PMC5889581; doi:10.1186/s12859-018-2113-6)
Supplement: Supplementary file 2 — Software build. Executable binary file of the software tool. (ZIP 118,784 kb) [file 12859_2018_2113_MOESM2_ESM.zip › cozoid/java/docs/beansbinding-1.2.1-doc.zip!/org/jdesktop/el/impl/parser/package-summary.html]

org.jdesktop.el.impl.parser (Beans Binding)


---


|  |  |  |
| --- | --- | --- |
| **Overview** \| **Package** \| Class \| **Use** \| **Tree** \| **Deprecated** \| **Index** \| **Help** | |  |
| **PREV PACKAGE**   **NEXT PACKAGE** | **FRAMES**    **NO FRAMES**     **All Classes** |  |


---

## Package org.jdesktop.el.impl.parser

Customized version of EL for Beans Binding; not for general use.

**See:**
  
          **Description**

| **Interface Summary** | |
| --- | --- |
| **ELParserConstants** |  |
| **ELParserTreeConstants** |  |
| **Node** |  |
| **NodeVisitor** |  |

| **Class Summary** | |
| --- | --- |
| **ArithmeticNode** |  |
| **AstAnd** |  |
| **AstBracketSuffix** |  |
| **AstChoice** |  |
| **AstCompositeExpression** |  |
| **AstDeferredExpression** |  |
| **AstDiv** |  |
| **AstDotSuffix** |  |
| **AstDynamicExpression** |  |
| **AstEmpty** |  |
| **AstEqual** |  |
| **AstFalse** |  |
| **AstFloatingPoint** |  |
| **AstFunction** |  |
| **AstGreaterThan** |  |
| **AstGreaterThanEqual** |  |
| **AstIdentifier** |  |
| **AstInteger** |  |
| **AstLessThan** |  |
| **AstLessThanEqual** |  |
| **AstLiteralExpression** |  |
| **AstMinus** |  |
| **AstMod** |  |
| **AstMult** |  |
| **AstNegative** |  |
| **AstNot** |  |
| **AstNotEqual** |  |
| **AstNull** |  |
| **AstOr** |  |
| **AstPlus** |  |
| **AstString** |  |
| **AstTrue** |  |
| **AstValue** |  |
| **AstValue.Target** |  |
| **BooleanNode** |  |
| **ELParser** |  |
| **ELParserTokenManager** |  |
| **SimpleCharStream** | An implementation of interface CharStream, where the stream is assumed to contain only ASCII characters (without unicode processing). |
| **SimpleNode** |  |
| **Token** | Describes the input token stream. |

| **Exception Summary** | |
| --- | --- |
| **ParseException** | This exception is thrown when parse errors are encountered. |

| **Error Summary** | |
| --- | --- |
| **TokenMgrError** |  |

## Package org.jdesktop.el.impl.parser Description

Customized version of EL for Beans Binding; not for general use.
It is expected that we'll sync up with an official version of EL
in the future.

---


|  |  |  |
| --- | --- | --- |
| **Overview** \| **Package** \| Class \| **Use** \| **Tree** \| **Deprecated** \| **Index** \| **Help** | |  |
| **PREV PACKAGE**   **NEXT PACKAGE** | **FRAMES**    **NO FRAMES**     **All Classes** |  |


---
